# Deformation mechanics of non-planar topologically interlocked assemblies with structural hierarchy and varying geometry

**DOI:** 10.1038/s41598-017-12147-3

**Published:** 2017-09-19

**Authors:** Lee Djumas, George P. Simon, Yuri Estrin, Andrey Molotnikov

**Affiliations:** 1 Department of Materials Science and Engineering and New Horizons Research Centre, Monash University, Victoria, 3800 Australia; 2 Laboratory of Hybrid Nanostructured Materials, National University of Science and Technology “MISIS”, Leninsky prospect 4, 119049 Moscow, Russia

## Abstract

Structural hierarchy is known to enhance the performance of many of Nature’s materials. In this work, we apply the idea of hierarchical structure to topologically interlocked assemblies, obtained from measurements under point loading, undertaken on identical discrete block ensembles with matching non-planar surfaces. It was demonstrated that imposing a hierarchical structure adds to the load bearing capacity of topological interlocking assemblies. The deformation mechanics of these structures was also examined numerically by finite element analysis. Multiple mechanisms of surface contact, such as slip and tilt of the building blocks, were hypothesised to control the mechanical response of topological interlocking assemblies studied. This was confirmed using as a model a newly designed interlocking block, where slip was suppressed, which produced a gain in peak loading. Our study highlights the possibility of tailoring the mechanical response of topological interlocking assemblies using geometrical features of both the element geometry and the contact surface profile.

## Introduction

The concept of *topological interlocking* ^1^ has emerged as a novel approach to meeting the challenges faced in materials science and engineering, due to its ability to combine useful mechanical and functional properties^2,3^. The concept is based on the segmentation of monolithic materials into specially designed, discrete identical units which interlock in three dimensions to form an assembly^4^. In this way, each element is constrained by the geometry of its neighbouring elements, providing the assembly a mechanism to have load-bearing structural integrity without the need for connectors or binders. A global constraint in the form of a frame, corner fasteners or tensioned wires is required to maintain the overall structure^5^.

These systems have been found to possess a range of interesting and useful mechanical properties. Damage tolerance^6^, bending compliance^7^, structural stability, and energy absorption^8^ are some of the key properties that are enhanced due to the inability for cracks to propagate across the boundaries of the discrete constituent elements.

The concept of topological interlocking materials based on this design principle was most clearly defined, in an engineering sense, by Dyskin *et al*.^4^ and Estrin *et al*.^1^, with its mathematical foundations presented in^9^, and has substantially advanced in recent years. There are unexpected similarities of the principle of topological interlocking with earlier concepts in masonry dating back hundreds of years, including the design of Truchet’s and Abeille’s vaults, as discussed by Fallacara^10^. The work by Glickman in the field of civil engineering^11^ predated the fully fledged development of this concept in the materials engineering context. The design principle based on topological interlocking has subsequently been applied to a range of areas, including sound absorption^12^, sandwich panels^13^, impact mechanics^14^, adaptive structures^15,16^, multi-materials composites^17^, and architectural design^10,18,19^. A recent article by Siegmund *et al*.^20^ provides a comprehensive summary of this field.

Various geometries of elementary building blocks that permit topological interlocking have been identified, cf., e.g.^1^. One particular family of such geometries are platonic bodies^5^. Examples of structures of this kind assembled from cubes and tetrahedra have been well studied^9,21–23^, with thrust-line models established^24^ and stiffness-scaling laws developed^25^. The deformation mechanics of assemblies of topologically interlocked blocks has been discussed by Dyskin *et al*.^26^ and Khandelwal *et al*.^24^. It has also been considered in detail by Mirkhalaf *et al*.^27^, who identified three primary sources of deformation and failure: (i) tilt of the assembly, (ii) slip or sliding of the central element under a concentrated load, and (iii) local irreversible (plastic) deformation of the central element and those in its vicinity.

**Figure 1:**
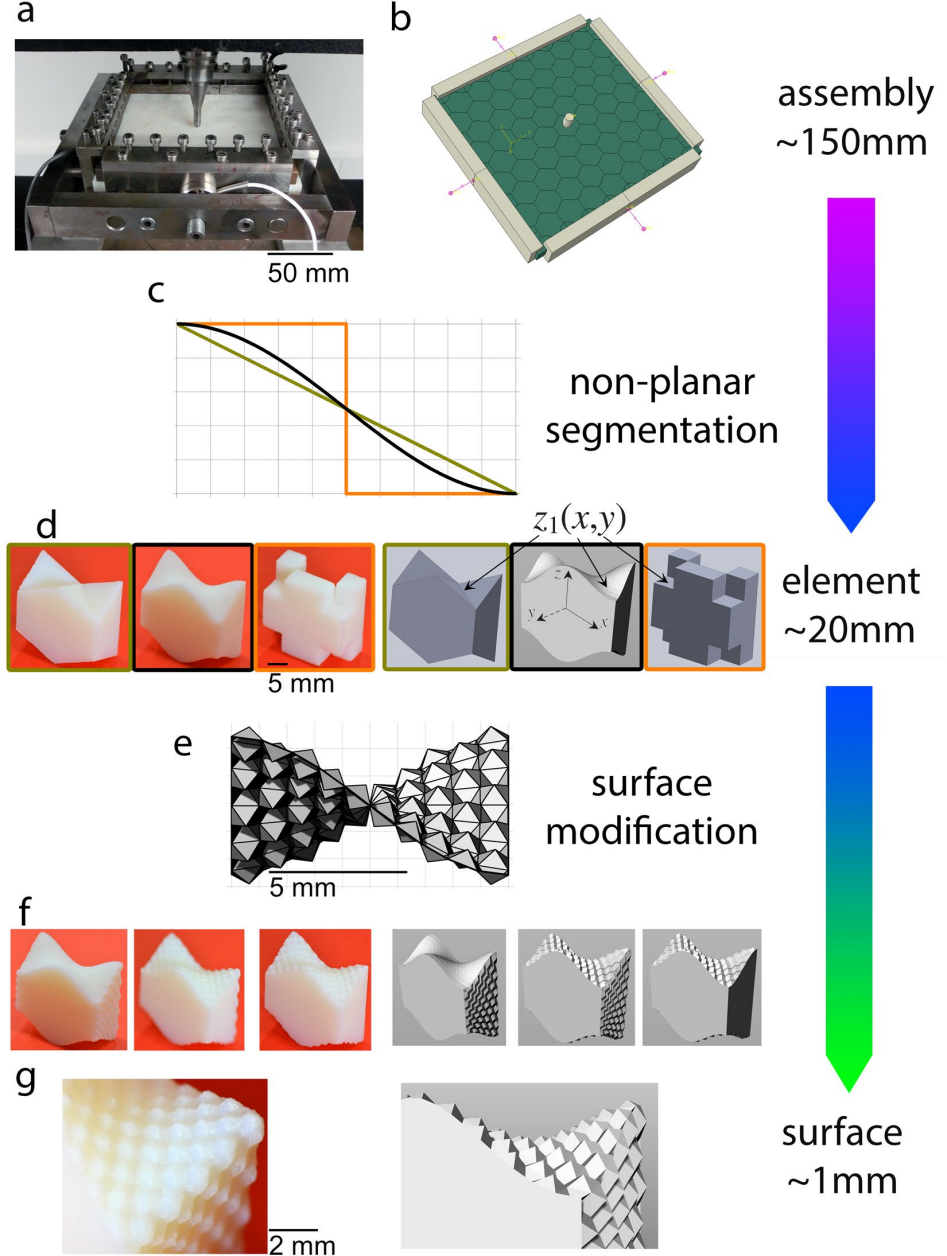
Overview of the hierarchical approach to block geometries and piecewise planar block assemblies used – (**a**) experimental setup showing assembled osteomorphic blocks, indenter, screw-driven frame and lateral force sensors. (**b**) Computational setup with the same dimensions as the experimental one (160 mm × 150 mm) and the block size of 20 mm × 20 mm × 10 mm. (**c**) Two-dimensional representation of shapes used for non-planar interlocking curves for block geometries, orange: square, black: osteomorphic, dark yellow: triangle. (**d**) As-fabricated and designed individual blocks with various geometries. (**e**) Representation of pyramidal surface modifications to an osteomorphic block. (**f**) As-fabricated and designed individual blocks, with various hierarchically interlocked surfaces. (**g**) Pyramidal profile design, individual pyramids approx. 1–2 mm in size.

Topologically interlockable elements with non-planar surfaces in the form of *osteomorphic* blocks, so named due to their similarity to the shape of bone, have also been studied [5]. These blocks interlock due to their matching concavo-convex surfaces, which are defined by a set of mathematical equations. The matching surface can be described as following:

1 $${{\rm{z}}}_{1}({\rm{x}},{\rm{y}})={\rm{\Delta }}{\rm{h}}\,{\rm{f}}({\rm{x}})\,{\rm{f}}({\rm{y}})+{\rm{h}},$$

where the function *f* meets certain periodicity conditions and Δh and h control the degree of interlocking, see Fig. 1d ^6^.

Studies in the past have used different functions for these elements, including fourth order polynomials^6^, a cosine function^17^, as well as “circular arc” and “straight edge” (triangle)^28^, all of them satisfying the symmetry and periodicity conditions required for interlocking^6^.

The concept of segmentation, often in conjunction with interlocking of the building blocks of a structure, can be observed in Nature^29^, and some prominent examples include nacre^30,31^ and red slider turtle carapaces^32,33^. The architecture of both nacre and turtle suture combines segmentation into interlocked elements with a hierarchy of structural features at multiple length scales^34,35^. A biomimetic approach adopting these concepts for use in engineering structures has driven scientists and engineers to develop new structural materials with growing success^36,37^. Areas of particular interest include lattice structures^38,39^, bio-inspired composites^40–43^, spider silk^44^, and smart actuators^45^. Structural hierarchy and interlocking in two dimensions have also been investigated, providing evidence that length hierarchy in a bio-inspired suture joint geometry gives rise to improved mechanical behaviour^46–48^. Niebel *et al*.^49^ also used hierarchical surface roughening to improve the ductility and fracture toughness of nacre-inspired structures.

In this work, we apply the idea of hierarchical structure to topologically interlocked assemblies and study the behaviour of such assemblies under point indentation load. We developed a specific script in the Rhinoceres CAD package which allows an *ad hoc* creation of hierarchical features on any surfaces of the interlocked blocks. We hypothesise that the mechanical response of topological interlocking assemblies is controlled by geometry of the interlocking elements and their surfaces as well as local surface patterns that may be introduced additionally. The deformation mechanics of these structures were revealed using finite element analysis, which provided us with an insight into different mechanisms of interaction of the surfaces such as tilt, slip, and plastic deformation. The simulation results were validated through experimental work. A particular focus of this study was to evaluate the effect of a secondary profile introduced on the interlocking contact surfaces of osteomorphic blocks and to understand the link between the elementary deformation mechanics of the blocks within an assembly and the mechanical response of the latter in terms of the load vs. displacement curves. Using the obtained knowledge two new interlocking block geometries were derived from the original design of osteomorphic blocks. It was shown that the mechanical response of the assemblies under point loading was a direct result of changes in the deformation mechanics of the blocks. This recognition provides possibilities of altering the mechanical behaviour required for specific applications through block geometry control.

## Results

### Design of structures

In this study, a well-established, previously reported osteomorphic block design with the curvature of the contact surfaces of the blocks defined by substituting the f(x,y) term with a cosine function (Fig. 1)^6,26,28^ and Δh and h set to 2.5 mm and 7.5 mm, respectively. These non-planar, curved surfaces lead to a large (as compared to basic polyhedra), contact surface area between elements, as well as minimising stress concentrations within an element^6^. It should be noted that in the mentioned studies a fourth order polynomial, rather than a cosine function, was used. However, both functions generate similar curved surfaces, and the small differences between the two geometries did not lead to any appreciable differences in the mechanical properties of the assemblies^28^.

This work involved two major transformations of the basic osteomorphic element, which is considered as a reference element: modifications to the surface to enable hierarchical interlocking, and changes to the geometry of the element itself. Implementing these design concepts involved writing a custom script in the Rhinoceres 3D 5.0 (Robert McNeel & Associates) CAD package to create a fully parametrised design of these complex geometries, which allowed significant freedom over the scale of various features.

The first modification involves changes to the morphology of the surface to add an additional degree of interlocking at a length scale one order of magnitude lower than interlocking at the scale of the blocks themselves. The reference element size has a centimetre length scale, while the secondary surface features introduced here are at a millimetre length scale. These surface modifications are inspired by the suture joints of red-eared slider^33^ and leatherback^50^ turtle carapaces. In these shells, interdigitated features provide the ability to reconcile the conflicting properties of flexibility and rigidity, providing the one or the other as required. In this work, we modify the primary interlocking surfaces by establishing a periodic pattern of pyramids with 1 mm × 1 mm base and 0.5 mm height, Fig. 1c and d. In this pattern, outward and inward facing pyramids alternate. Due to this secondary surface feature, the matching faces of the neighbouring blocks will interlock at a new length scale, in addition to being interlocked at the scale of the entire block itself. Indeed, this design also increases the number of interacting surfaces by a factor of approximately 500 and the overall surface area by approximately 20%. Importantly, the size scale at which this feature occurs - an order of magnitude smaller than the element size - is still sufficient to observe geometrical interlocking, as opposed to the inherent surface roughness at the level of random, irregular asperities. This addition of a secondary interlocking pattern at the surface of the blocks to the primary interlocking of the blocks will thus be referred to as hierarchical interlocking.

The second variation involves a change in the element geometry. The cosine osteomorphic element was again used as a reference, while two contrasting surface geometries were developed to compare the deformation mechanics of the assemblies based on such elements. While previous reports focused on some changes to the surface curvature^28^, or changes to tetrahedron-shaped elements^27^, here we present a triangle curve and a square curve as more extreme examples of interlockable blocks with piecewise planar surface geometries, cf. Fig. 1d. These curves follow the same mathematical formulation as the original osteomorphic blocks, except with the cosine function being replaced by a triangular wave function and a square wave function. A geometry similar to the triangle one has been previously reported^23,28^, and the building block of this kind will be referred to as a “triangular element”. A contrasting geometry is represented by the square curve, Fig. 1d. It will be denoted as the H+ element due to the letter “H” shape being apparent when viewed from one of its sides, and the “plus” sign visible from the opposite side. We hypothesise that this element will completely suppress the ability for the elements to slide or slip past each other, which is useful for analysing the effect of slippage on the mechanical performance of topological interlocking assemblies. In contrast, the triangle elements are expected to allow for more slip than the cosine osteomorphic elements, due to their piecewise flat surfaces. Indeed, the straight triangle elements bear a resemblance to tetrahedron-shaped elements, and it could be anticipated that the mechanical performance of an assembly of triangle elements would be similar to that of tetrahedra. This provides a convenient way of comparing the different geometries, while maintaining similar size parameters of the elements.

Below, we look into the key mechanics underlying the mechanical response of this class of architectured materials with the aim to provide some guidelines for future design of topological interlocking materials.

### Hierarchically interlocking assemblies

**Figure 2:**
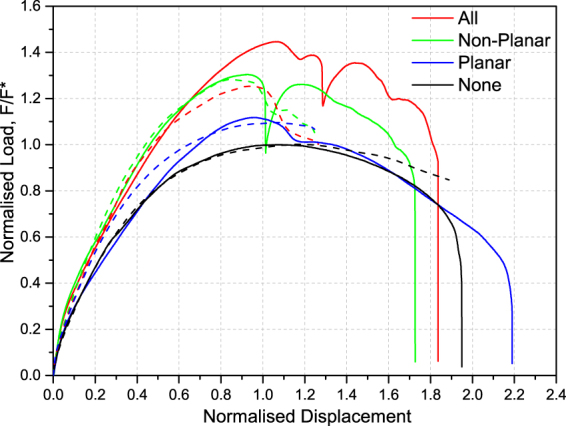
Load-displacement curves for hierarchically interlocked assemblies. Load F is normalised with respect to the peak load F* for the assembly of reference osteomorphic blocks, while displacement is normalised with respect to the plate thickness. The solid lines represent experimental data and the dashed lines represent computational results, which are presented up to the displacements at which a divergence from the experimental results occurs. Experimental testing was conducted in triplicate as a minimum.

In order to study the effect of hierarchical interlocking, the pyramidal surface modification was applied to different surfaces of the osteomorphic element – either the curved interlocking surfaces or the planar side surfaces, or both, as shown in Fig. 1. The load-displacement curves (with load normalised to the peak load of the reference assembly and displacement normalised to the element thickness) of these hierarchically interlocked assemblies are summarised in Fig. 2, which reveals an interesting trend.

**Figure 3:**
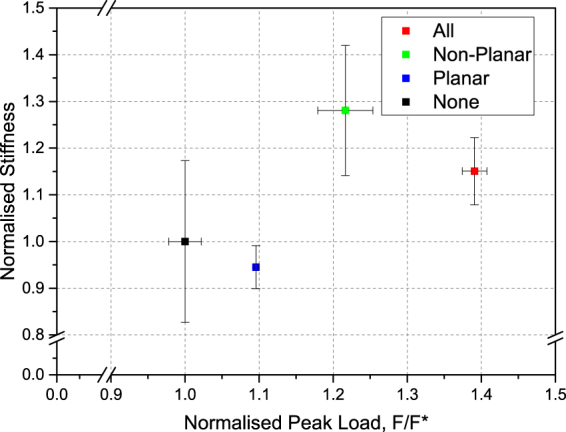
Experimental peak load vs. stiffness for assemblies with hierarchical interlocking, normalised with respect to the peak load and stiffness of a reference assembly of osteomorphic blocks. The error bars indicate the scatter in the data. Peak load error bar for the case referred to as “planar” are small and are contained within the symbol. Stiffness is taken as the tangent at 0.01 normalised displacement. Experimental testing was conducted in triplicate as a minimum.

It can be seen that the additional degree of interlocking, whilst maintaining a reasonably similar initial stiffness of the assemblies due to the secondary surface profile, led to an increase in the peak load in all cases (Fig. 3). The smallest increase in peak load was found when only planar side surfaces were modified by secondary patterning, and it was greater when the non-planar interlocking surfaces were patterned. The greatest effect was observed for the case of all surfaces being patterned. The occurrence of sharp load drops around or after the peak load appears to coincide with a sudden slip of the central loaded element, relative to the neighbouring elements, due to the surface pyramids sliding past one another. This “softening” of the assembly tends to follow these sudden decreases in load before failure. A smaller irregularity, possibly due to slip of the block under load as well, is observed for the reference sample at a lower deformation (normalised displacement of about 0.3).

The above idea that the precipitous load drops are the result of slip of the central element relative to the rest of the assembly suggests that the peak load can be increased by inhibiting the onset of slippage. Slip also leads to a decrease in the deflection at failure, cf. Fig. 2. This minor decrease is most likely related to breakage of the surface pyramids at the edge of the central element, followed by slip of the central element as a whole. Another contributing mechanism is the “tilt” of the entire assembly as point loading occurs. These two effects are not mutually exclusive and occur in parallel, as discussed by Mirkhalaf *et al*.^27^, and they were also observed for the reference osteomorphic assembly. However, it is likely that tilt can be more closely related to the ascending, ‘elastic’ portion of the curve, while slip of one or more blocks signifies the onset of the irreversible, ‘plastic’ deformation.

Initial observation of the results seems to indicate that the surface patterning producing an extra level of interlocking simply gives rise to increased friction. There are a few important differences between the hierarchical interlocking and friction. Conventional friction is generally associated with surface roughness, primarily due to random, irregular asperities at the nanometre to micrometre length scale. Discontinuous sliding referred to as “stick-slip” can occur when in a certain range of sliding velocities, the coefficient of friction is a decreasing function of the velocity. This may be a result of a negative rate sensitivity of the asperity shearing or breakage^51^. The interdigitated pyramidal features imposed in this work are regular, ordered and designed to interlock at a millimetre length scale. Indeed, here we propose that the large spasmodic drops in load observed in Fig. 2, are not due to frictional stick-slip, but are rather associated with the pyramids on the mating surfaces sliding past one another in a collective process. This conjecture is supported by the fact that almost no visible damage or shearing of the surface pyramids was observed ‘post mortem’. This behaviour was also confirmed by the numerical simulations. Our current view is that the observed results stem from a combination of an increase in the contact area between the blocks and the extra resistance to sliding due to a large number of pairs of pyramids on the mating surfaces engaged in interactions at a multitude of angles.

The behaviour of assemblies with the four geometries considered was simulated using FEA to gain a better understanding of the response of the structures to the point loading. Figure 2 shows a good qualitative and quantitative agreement with experimental results. Initial stiffness is well matched for all samples, excluding the example of planar which slightly over-predicts stiffness, and the magnitude and location of the peak loads are also in good agreement. The complex geometry of the full hierarchical specimen (designated ‘All’) resulted in computational difficulties, leading to lower-strain divergence from experimental results. Interestingly, the model does predict a significant drop in peak loads around 1.0 normalised displacement in both ‘All’ and ‘Non-Planar’ samples with good accuracy, capturing the effect of neighbouring surface pyramid sliding over one another.

**Figure 4:**
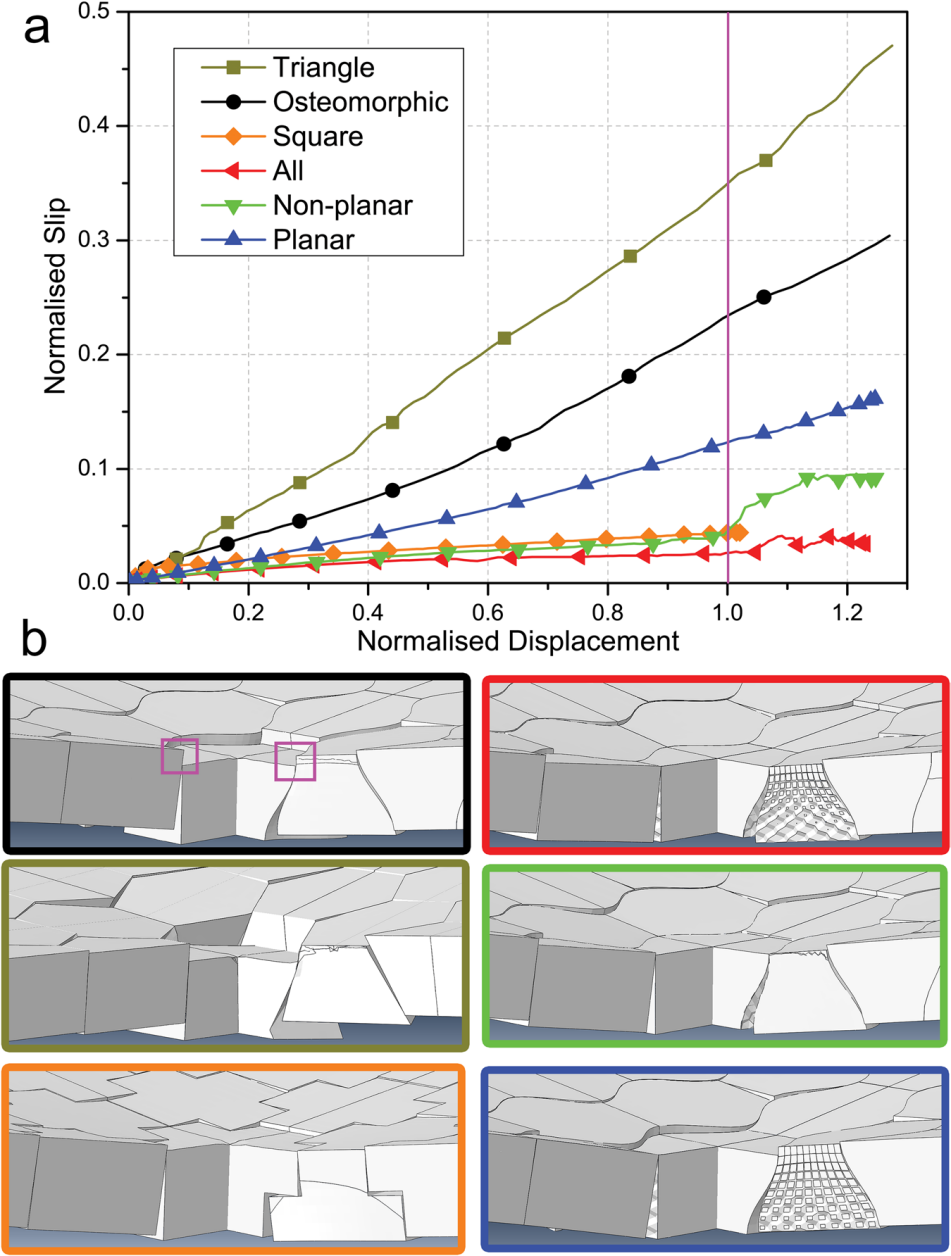
Displacement of central element relative to neighbouring elements as measured by average distance of two edges shown in purple squares: (**a**) plotted as a function of normalised displacement, (**b**) images of computational output for central element at 1.0 normalised displacement (as shown by the purple line in Fig. 4a). Colour borders correspond to curve colours in Fig. 4b. Displacements have been normalised in the same way as in Figs 2 and 5.

The assemblies were inspected visually and simulated numerically, by finite element analysis (FEA), with particular emphasis on the behaviour of the central elements during deformation. The amount of slip (the relative displacement of the central element to its directly neighbouring elements) is shown as a function of the overall normalised displacement for the various geometries (including those discussed in the section below) in Fig. 4a. This provides a quantifiable measurement of the contribution of slip to the overall displacement of the assembly, with the other contributions being assembly tilt and plastic deformation. This plot demonstrates that geometries which have higher load bearing capacity slip less than those with a lower load bearing capacity. Indeed, if we take the point at normalised displacement of 1.0, we observe that the reference assembly exhibits approximately 0.23 slip, the planar one 0.12, and the non-planar one 0.04, while the ‘All’ assembly experienced less than 0.03 slip. The difference between these values and the magnitude of total normalised displacement (1.0) is the contribution of tilt and/or plastic deformation to the overall displacement. These values show a clear correlation between load and slip. Indeed, a significant increase in slip is observed between 1.0 and 1.2 normalised displacement, which corresponds well with the decrease of load observed in the curves presented in Fig. 2.

The different slip characteristics of the assemblies considered were evaluated by FEA at 1.0 normalised displacement. They are visualised in Fig. 4b. A large displacement of the central element of the reference geometry and a negligible displacement for the ‘All’ geometry can both be seen. The simulation results for non-planar and planar element assemblies display the effect of the surface modifications truthfully, the planar hierarchically interlocked surface interaction showing minimal slip and the curved concavo-convex surface exhibiting the ability to slip.

### Modified geometries to adjust slip characteristics

**Figure 5:**
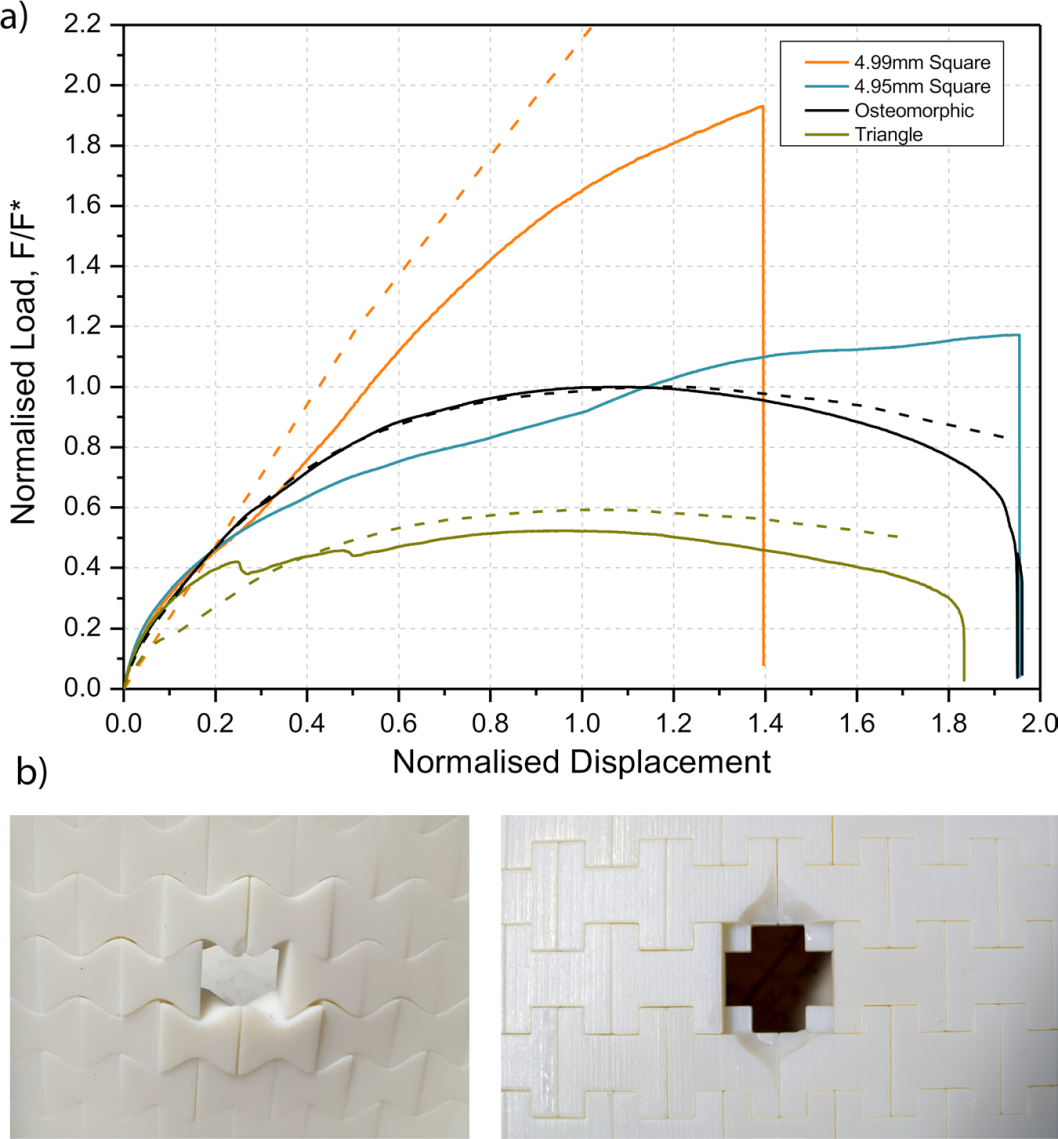
(**a**) Normalised load-displacement curves for assemblies of interlocked elements with non-planar surfaces. The load and the displacement have been normalised in the same manner as in Fig. 2. The solid lines correspond to the experimental curves and dashed lines to the computational curves. (**b**) Damage to osteomorphic and square assemblies after mechanical testing.

A comparison between the load vs. displacement curves of three primary shapes - smooth (cosine), straight triangular, and square (H+) blocks is shown in Fig. 5a. Once again, the results of cosine-generated osteomorphic element assembly are included as a reference. One of the primary effects observed in Fig. 5a is the shape of the curves for the H+ blocks (4.99 mm and 4.95 mm in size), which is distinctly different to that for the reference assembly and the assembly of straight triangle elements. It is clear that the H+ elements show no ‘deformation softening’, the load increasing continuously until brittle failure, which then occurs suddenly and without warning. By contrast, for the assembly of straight triangle elements, a decrease in the peak load, compared to the reference assembly, can be observed. Indeed, the load vs. displacement curve departs from the linear elastic region at relatively low deflections, which may be attributed to the greater ability of the central element to slip relative to the surrounding elements of the assembly.

**Figure 6:**
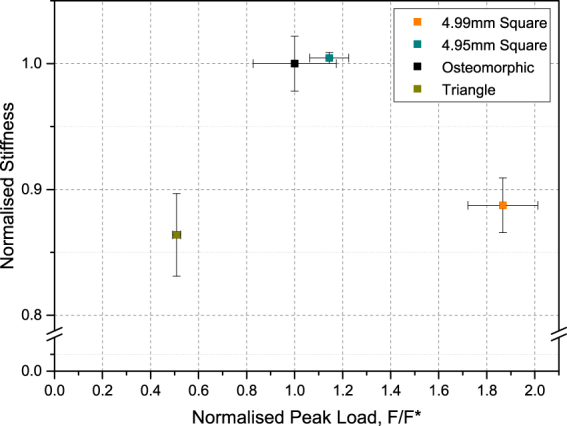
Experimental peak load and stiffness for assemblies with modified geometry, normalised with respect to the peak load and stiffness of a reference assembly of osteomorphic blocks. The error bars indicate the scatter in the data. Stiffness is taken as the tangent at 0.01 normalised displacement.

For all samples, nearly the same initial region (up to about 0.12 normalised displacement) is observed. This indicates that loading at low strains is not geometry- dependent (as observed earlier for the hierarchically interlocked samples). More likely the initial response to loading is governed by other variables, such as element size, magnitude of the coefficient of friction, material properties, and boundary conditions, as shown in previous studies by Schaare *et al*.^8^ and Khandewal *et al*.^8,25^. It is only after this initial deformation stage that the geometry becomes critical. This effect is demonstrated in Fig. 6, which shows a similar initial stiffness for the different assemblies tested, while displaying varying peak loads.

It appears that after the initial stages of displacement, in the ca. 0.2 normalised displacement region, the effects of sliding and tilting become important to various degrees dependent on geometry. Around this transition region, the load on the H+ assembly continues to rise, and presumably only tilting occurs. The load on the reference osteomorphic assembly is also still increasing, however at approximately 0.25 normalised displacement this growth begins to slow down, as the sliding sets in and acts concurrently with tilt. Finally, the triangular element assembly exhibits a significant load decrease at around 0.25 normalised displacement, and another at 0.5 normalised displacement. These drops in load appear to be related to the slip mechanism discussed above, and their presence seems to correspond to a transition from static to kinetic friction – a hypothesis that would require further study to confirm. The load-displacement curve of the triangle geometry is much flatter and has a lower peak load compared to the assemblies with the other two geometries. From a value of the normalised displacement approximately in the range of 0.4–1, the H+ element assembly keeps showing an increase in load, while significant deformation softening is seen for the other two geometries, with the triangle element assembly reaching its peak load at 0.95 normalised displacement - a slightly lower value than the one for the reference, cosine element assembly (1.05).

After a peak, a gradual decrease in load is observed for the osteomorphic and straight triangle element assemblies until their failure occurs at 0.195 and 0.183 normalised displacement, respectively. In contrast, the H+ assembly fails in a more brittle manner (Fig. 5b). The point at which failure occurs depends upon the tightness of fit (4.99 mm vs. 4.95 mm block size), as does the peak load and the accompanying displacement. The brittle failure of the H+ assembly was found to be confined to five elements in the middle (the central element and the four adjacent ones). A greater amount of local plastic deformation was also detected in the H+ elements at the indentation point on the central element and its neighbours as compared to other assemblies (Fig. 5b). In addition to increased peak load, enhanced energy absorption or toughness modulus, as represented by the area below the load-displacement curve, was observed for the two H+ element assemblies. This might be associated with increased plastic deformation or propensity for damage of the elements – a proposition that needs to be tested in future experiments on different materials. We note, however, that elements of the osteomorphic and straight triangle assemblies exhibited only small amounts of damage at failure (Fig. 5b).

Figure 5a also shows computational results for the three different geometries considered. The experimental and computational results for osteomorphic element assembly are in very good agreement and diverge only slightly towards final failure, likely due to the exclusion of damage and plasticity from the simulation. The results for the square element assembly also provide reasonable agreement, the simulation slightly overestimating the load, again most probably due to some plastic deformation of the material not considered in the simulations. Finally, there is a greater discrepancy between the computational and experimental results for the triangle element assembly. This could be attributed to differences in the interface behaviour, primarily the difference in static and kinetic friction occurring at a normalised displacement of about 0.1, the simulation displaying a more “ideal” scenario with negligible static friction. Overall, the simulations agree well with the experiments, qualitatively and semi-quantitatively, which means that they capture well the mechanics of the assembly, as shown in Fig. 4a. Furthermore, it should be noted that the simulations are the only tool for validating our hypotheses related to different mechanisms of interaction of the interfaces.

The systems with modified geometry are also included in Fig. 4, which displays the slip behaviour of the central elements under loading based on FEA results. We observe similar trends to those stated in the foregoing section, with a clear correlation between load bearing capacity and slip. In Fig. 4a, at normalised displacement of 1.0, we observe that the osteomorphic sample has experienced approximately 0.23 slip while the triangle assembly has undergone 0.35, and the square sample less than 0.05 slip. Additionally, the images presented in Fig. 4b show visually the different slip characteristics of the different assemblies and exhibit a large displacement of the central element observed in the triangle and osteomorphic assemblies at 1.0 normalised displacement. By contrast, the square sample shows negligible slip at this displacement.

The results presented here show that by manipulating the ability of the constituent elements of topological interlocking assemblies to slip relative to one another, better control of the mechanical response of this class of materials can be obtained.

The hierarchical interlocking results suggest that the effect of slip is critical to the overall performance of the assembly, as it appears that by delaying the onset of significant slip, the overall peak load can be raised. This hypothesis was tested by designing elements (H+ blocks) that would supress slip entirely. Indeed, we observed that no softening of topological interlocking assemblies occurs when slip is absent, hereby directly linking softening with slip in topological interlocking assemblies for the first time. This is an important finding, and will enable better design of these types of structures moving forward.

Our experimental and computational data also agree well with the previously reported results by Khandewal *et al*.^25^, where the initial stiffness of topological interlocking assemblies was studied under suppression of slip. Furthermore, the ability to modify the displacement at which peak load occurs is also demonstrated here. Previous studies by Brugger *et al*.^52,53^ and Duguè *et al*.^23^ refer to a “master curve” where for a fixed geometry the same shape of the load-displacement curve is achieved, regardless of variations of material properties, element size, friction, etc. The present study highlights the ability to modify this behaviour by *geometry variations*.

An important outcome of our study is the recognition that the mechanical response of ensembles of topologically interlocked blocks is determined by different modes of block interaction, including tilt, slip, and plastic deformation, and that the relative contributions of these modes depend on the block geometry.

## Conclusions

This work provides crucial insight into the mechanical response of topological interlocking assemblies under point loading. The key outcomes of the work can be summarised as follows.

- A secondary surface geometry was introduced to non-planar elements to create an additional interlocking effect between neighbouring elements at a new length scale, referred to here as hierarchical interlocking.
- Experimental results suggest that the hierarchical features associated with patterning of the interlocking block surfaces cause a delay of the onset of slip of the central block under concentrated load and the attendant increase in the maximum load bearing capacity of the overall assembly.
- This concept was further tested by designing and testing new topological interlocking elements, termed the H+ element, which prevented slip. The tests showed that without slip, topological interlocking assemblies could not “soften” – linking the two mechanisms for the first time.
- Computational results demonstrated that the slip mechanism is correlated with the mechanical behaviour of these geometrically complex assemblies.

In summary, this study showed that geometrical modifications to the original osteomorphic element and patterning of the element surfaces provided a way to manipulate the deformation mechanisms of topologically interlocking assemblies, thereby controlling the mechanical response under point loading. These new insights gained from the computational modelling and experimental data enable design of topological interlocking structures with tailored mechanical properties in the future.

## Methods

### Fabrication

The specimens were fabricated using an Objet Connex500 multi-material inkjet 3D printer manufactured by Stratasys Ltd. It employs polymer jetting technology which dispenses a photopolymerisable monomer resin which is subsequently cured with ultraviolet (UV) light and solidifies in a layer-by-layer fashion. The material used has a commercial name of VeroWhitePlus (VW+) which is a stiff/rigid proprietary blend of acrylic-based polymer. The inkjet technology is a versatile tool that provides a possibility to print complex, multi-material and high-resolution specimens. This technology has been employed in a number of studies of the mechanical response of architectured materials^58,40,54–57^. Figure 1 shows the fabricated and designed individual blocks and block assemblies, with various hierarchically interlocked surfaces. The size scale of the secondary surface pattern was selected to be in sub-millimetre range which is linked to the precision of the 3D printer used. Although smaller features can be printed using the inkjet technology, our preliminary testing demonstrated that these features do not possess sufficient mechanical strength.

### Mechanical testing

The additively manufactured elements had dimensions of 20 × 20 × 10 mm^3^ assembled into plates with 160 mm × 150 mm dimensions and 10 mm thickness. The plate was held in a steel frame with adjustable screw-driven boundaries capable of controlling the lateral load. Mark-10 MR02-500 force sensor were utilised to measure the applied lateral pre-load and all tests were conducted under 1 kN pre-load. A point load was applied perpendicular to the plate via a rounded steel indenter with 7 mm diameter attached to a 5 kN static load cell in an Instron 4505 testing machine at a constant speed of 4 mm/min (Fig. 1a.). At least three tests were conducted for each type of assembly.

### Computational work

Finite Element Analysis (FEA) was utilised to study the behaviour of interlocked structures under point loading and is essential for understanding the interaction of adjacent blocks as well as the combined behaviour of the assembly. ABAQUS/Explicit v6.14 solver was selected due to its ability to deliver a convergent solution for highly nonlinear systems with many complex surface interactions under transient loads. To model the contact between elements, a general contact algorithm was used as a penalty method (a stiffness based approach). The coefficient of friction was set to 0.3, based on experimental work conducted by our group. The assemblies were assumed to be under quasi-static loading, which represented a challenge to the solver’s efficiency. In order to accelerate the quasi-static simulations, the time step was reduced artificially. The validity of this approach was examined by tracing the inertial forces and incrementally reducing the time steps until the results began to diverge, and become unrepresentative. The ratio of the kinetic energy to the internal energy for the shortest time increment providing accurate results was then calculated to ascertain that the kinetic energy was sufficiently small (i.e. less than 1% of the internal energy)^59^. A loading time of 0.15 s used in the simulations was found to be adequate.

Mesh convergence studies were carried out to determine the necessary element size. A mesh density of approx. 3800 elements per block was found to be sufficient to accurately represent stresses with adequate resolution within a block.

The assembly was defined as an isotropic elastic material with values for Young’s modulus (1700 MPa), Poisson’s ratio (0.39), and density (1.17 g/cm^3^) derived from our mechanical experiments. Similar values were reported by others^58^. Plasticity and damage of the material of the blocks were disregarded in this simulation. Therefore, it is expected that the simulation results may diverge from the experimental results when approaching the final failure of the assembly or upon complete block removal.

The point indenter and the frame were modelled as rigid bodies to which external loads were applied. In the case of the point indenter, a smooth step displacement was applied to the top of the indenter at which point the force was measured. The force required to model the pre-load of the assembly and the associated elastic compliance was modelled using a single spring element attached to the rigid body frame, Fig. 1b. A similar method whereby the spring element was displaced to apply lateral pre-load was previously employed by Schaare *et al*.^8^. This is the only fitting parameter of the simulation and to replicate the 1 kN lateral pre-load of the experimental set-up, a spring stiffness of 2 kN which was displaced by 0.05 mm was found to give the best fit to the elastic compliance of the control assembly. This set up was adopted for various assembly geometries.

In order to model the highly complex hierarchical interlocking elements, a multi-stage process was utilised. This involved generating an appropriate mesh using HyperMesh (part of the HyperWorks suite, Altair Engineering, Inc.) of approx. 12,000 elements per block. The obtained mesh was imported into the ABAQUS model as described above, with the central 7 elements replaced with the hierarchical interlocking meshed blocks. This approach is justifiable as it provided a sufficiently representative approximation to the experimental response, while maintaining a modest number of elements and therefore minimising computational time.

### Data Availability

The datasets generated and analysed during the current study are available from the corresponding authors on reasonable request.
